# Peer clustering of exercise and eating behaviours among young adults in Sweden: a cross-sectional study of egocentric network data

**DOI:** 10.1186/1471-2458-13-784

**Published:** 2013-08-28

**Authors:** Kieron J Barclay, Christofer Edling, Jens Rydgren

**Affiliations:** 1 Department of Sociology, Stockholm University, Stockholm, Sweden; 2 Department of Sociology, Lund University, Lund, Sweden

**Keywords:** Social networks, Egocentric data, Social clustering of health behaviours

## Abstract

**Background:**

Research suggests that the growing prevalence of obesity may be related to the influence of the health behaviours of peers. We look at clustering of exercise and eating behaviours amongst a previously unstudied group, young adults in Sweden. Previous research has mainly been conducted in the United States and Britain, countries that have relatively high rates of obesity.

**Methods:**

Using ego-alter dyads from the egocentric network data as the unit of analysis, we conduct logistic regressions to investigate the association between ego and alter exercise and eating behaviours.

**Results:**

Respondents have a significantly greater probability of engaging in regular exercise and eating healthily if a nominated peer also does so. Furthermore, the degree to which this behavior is shared is modulated by the strength of the relationship between the two individuals, with a greater probability of engaging in these behaviours observed when the relationship with the nominated peer is strong relative to when the relationship is weak. However, we find that ego-alter homogeneity in terms of gender and migration status was not associated with a significantly greater probability of behaving in a similar manner to a nominated peer. Furthermore, the status of the nominated peer as a relative or not did not impact the probability that the ego would engage in similar health behaviours to that alter.

**Conclusions:**

We observe strong associations between ego and alter health behaviours for young adults, consistent with previous research. Although we cannot draw causal inferences, these results suggest that the health behaviours of an individual’s peers may play a role in shaping their own health behaviours.

## Background

Over the past thirty to forty years, there has been a secular increase in obesity across most of the world [1]. While the United States is the trend leader, this increase has also been observed in Europe, including Sweden [2]. The largest driver of this pattern has been a changing diet and an increase in sedentary behaviour [3,4]. International comparisons find that between 10% and 15% of adolescents in Sweden are classified as being overweight; a lower proportion than in the USA, UK, and South Western Europe, but more than in Eastern Europe [5], which suggests that Sweden performs relatively well. However, it can be argued that more research is needed in countries that are not experiencing shifts in obesity that are as dramatic as those seen in the USA and the UK [6-8]. It may be insightful to investigate the relative importance of peer influence in a less dramatic context, such as Sweden, and one that provides a clearer comparison to the experience of countries other than the United States.

Although the importance of changing environmental factors is clear, a growing body of evidence has demonstrated the degree to which the social network in which an individual is embedded is associated with that individual’s health and health behaviours [9]. In recent years several studies have investigated the social transmission of obesity [10-12]. However, despite the common discussion of obesity as an epidemic, the mechanism for this increase in prevalence is almost certainly changes in activity and consumption, as obesity is not a contagious pathogen. The explanation for its social transmission must lie in the realm of changes to attitudes and behaviour, and these are likely to be at least partly influenced by the preferences and actions of peers. The observation that changes in BMI over time are positively correlated across individuals who are connected is likely to be partially a function of a change in attitudes regarding the degree of acceptability of a given weight status [10]. However, even assuming a change in weight-related norms, for this to translate into an actual change in the ego’s own body, it must be mediated through some kind of behaviour, such as eating, exercising, smoking, or drinking.

### Explanatory mechanisms for observed social diffusion of behaviours

Previous research has suggested several different potential explanations to account for the observed diffusion of behaviours in a social network over time. These are selection effects, joint exposures, and induction. Selection effects refer to the assortative formation of relationships between similar individuals. Thus, individuals who are alike in terms of gender, educational level, socioeconomic background, and behaviours, such as exercising, will, *ceteris paribus*, be more likely to develop a relationship than individuals who are dissimilar in terms of these characteristics [13]. Joint exposures refer to a factor that is exogenous to the relationship itself between two people that could be responsible for them both gaining, or losing, weight. An example of this could be a fast food restaurant, or a gym, opening in the neighbourhood [14]. Finally, induction refers to a genuine peer effect, whereby the preferences or actions of a social contact, or alter, influence the preferences and actions of the focal individual, or ego [15]. While both selection effects and joint exposures are likely to play a role in the observation of the diffusion of health behaviours through social networks over time [16], the case for a genuine induction effect is growing, particularly in the light of experimental research showing evidence for peer influence [17-19]. However, we are not able to make causal inferences regarding the transmission of health behaviours between peers in this study as this causal relationship is not identifiable in the absence of full information concerning both the choice of friends and the proclivity to engage in a given health behaviour [20-22].

### Previous research on social transmission of exercise and eating behaviours

Although there is a substantial body of research emphasizing the importance of social support for an individual engaging in regular exercise [23-25], the volume of research using actual social network data is considerably smaller. It is important to distinguish between the social support literature and social network literature, as the latter allows for the investigation of how the specific characteristics of the ego-alter relationship and how certain characteristics of the alter are associated with the behaviour of the ego [9]. It is only within the past three years that studies have emerged using data on the specific exercise behaviour of alters [26-28]. Using longitudinal data from The National Longitudinal Study of Adolescent Health (Add Health) associations were found between the ego and alters engaging in regular exercise, and participating in sport [27]. Furthermore, a study using stochastic actor-based models for social network analysis [29], found that peer socialization was a key factor influencing ego physical activity, even after adjusting for factors that led individuals to select into these friendship relationships [28].

In terms of research investigating the degree to which ego and alter eating behaviour is associated, most studies have collected data about social support or perceived social influence from family or peers [30,31], while few have used actual social network data. One of the few studies to do so used longitudinal data from the Add Health project [27]. An association was found between ego and alter behaviour in terms of frequency of eating at fast food restaurants, but no statistically significant results were observed for eating breakfast regularly, consuming calorie-dense snacks, or consuming five portions of fruit and vegetables daily [27]. A similar result was observed in a study using the Framingham Heart Study data; Several different dietary patterns were identified through a combination of principal component factor analysis and cluster analysis, and it was found that the eating pattern most strongly associated between peers was primarily characterized by alcohol and snacks [32]. A study using cross-sectional network data collected in Australia found that male peers were likely to share a pattern of consumption of high calorie foods, though, again, the nature of the data did not allow for the authors to distinguish between selection effects and peer influence [26].

### Homophily and social influence

The assortative formation of relationships describes the tendency for similar individuals to form relationships with one another [13]. This similarity of social contacts, or homophily, also influences the degree to which individuals exert influence over one another in a manner that is distinct from homophilous tie formation, and also distinct from the process by which the relationship was originally formed [19,33]. For example, a male is more likely to influence the exercise patterns of another male, independent of the fact that they became friends partly because they were both males, and also independent of the fact that they met in a setting into which they both independently selected themselves due to a common interest. Research using experimental approaches provide convincing demonstrations of genuine induction effects [17-19,33], while evidence for homophilous influence is visible from the pattern that same-sex friends, and same-sex siblings, exert a greater influence than opposite-sex friends, and opposite-sex siblings [10,15], though this has been disputed [22]. Although past research has indicated that behaviour clustering amongst adolescents tends to be due to selection into groups with shared preferences in regards to the focal behaviour [34,35], results that indicate that the ego is more likely to behave in a certain way if the alter is similar to them in a number of different ways would suggest that homophilous influence is being exerted [36].

Studies have also found that the degree to which ego-alter health behaviours are associated is likely to be greater when the alter is a relative of the ego, such as a sibling [10,15,37]. These studies using the Framingham Heart Study data have also shown that the association between the alter’s obesity or smoking behaviour and the ego’s weight or smoking behaviour persisted regardless of the physical distance in geographical location between the two individuals. Although it is not possible to speak of a formal causal relationship, this does suggest a genuine induction effect. Given this precedent, we hypothesize that we will observe a similar pattern in this study: there will be a stronger association between ego-alter health behaviours for individuals who are similar to one another. We intend to test this by looking at ego-alter gender homogeneity, as well as whether the alter is a relative of the ego. Given that ego-alter homogeneity has been shown to be predictive of the spread of a behaviour between two individuals, we would also expect that there will be a stronger association between ego-alter health behaviours for individuals who are similar in terms of their migration background. In this case, migration background homogeneity refers to whether the ego and alter are first generation migrants, second generation migrants, or have two Swedish born parents.

### Aims

The purpose of this study is to investigate how ego health behaviours are associated with the health behaviours of their peers using a unique dataset containing egocentric network data on a cohort of young adults in Sweden. Two health behaviours will be investigated: healthy eating, and taking regular exercise. We will also investigate the degree to which the association between ego and alter behaviours is mediated by the strength of that relationship, as well as gender and migrant status homogeneity, and whether the ego and alter are relatives. This study will be the first to investigate the association of health behaviours between peers using social network data in the Swedish context. The particular virtue of conducting this analysis using a sample of young adults in Sweden is that health behaviour patterns established in adolescence and young adulthood are important for shaping long-term health trajectories by influencing both preferences and behaviour patterns [38]. Thus, research into the health behaviours of these young adults today will provide an early indication of the long-term trends that we can anticipate to observe for both this specific cohort, and the generation to which they belong. We will test the following hypotheses:

### Hypotheses

• H1: The ego will be more likely to engage in a given behaviour if the alter also does so.

• H2: The association between ego-alter behaviours will be greater the stronger the ego-alter relationship.

• H3: The association between ego-alter behaviours will be greater with ego-alter homogeneity

The first hypothesis, H1, describes that we expect that the ego will be more likely to exercise regularly if the alter also exercises regularly, and that they will be more likely to eat healthily if the alter eats healthily. This study will also address the extent to which both the strength of the relationship between the ego and alter (H2), and alter type (H3), interacts with this association. We expect that the degree of influence that the alter exerts will be greater the stronger the relationship is between the ego and alter. Alter type will be investigated in terms of gender homogeneity, migration background homogeneity, as well as whether the alter is a relative of the ego or not. We expect that when the ego and alter are of the same gender, same migration background, or are related, the alter should exert a greater degree of influence over the ego than would otherwise be the case.

## Methods

### Data

The data used for this study come from a survey conducted in late 2009. The survey sampled 5,695 Swedish youths aged 19, who were approached for a telephone interview by the government body Statistics Sweden between October and December. According to a consulting statement (2008/580-31 m), the Ethical Review Board (EPN) in Stockholm approved the ethical application. The sample is based on three different groups of Swedes, differentiated by the parental country of birth, born in 1990: (a) all individuals with at least one parent born in Iran, (b) 50 percent of all individuals with at least one parent born in the former Yugoslavia, and (c) a simple random sample of 2500 individuals with two Swedish born parents. The overall focus of the project for which this data has been collected is ethnic inequality, and the stratified sampling approach is explained by a desire to avoid collecting data on ‘immigrants’ who are highly heterogeneous as a group. A total of 2,942 interviews were conducted by Statistics Sweden, giving a response rate of 51.7 percent. The most common reason for non-response, 37.6 percent of the sample, was that the interviewers could not get in contact with the individual, while only 8.1 percent of the sample refused to participate. The main reason why it was so hard to establish contact was because of the prevalence of pay-as-you-go phone users in this age group. In these cases, names are not registered to particular phone numbers. The effective sample was slightly biased. Information provided by Statistics Sweden from the administrative registers showed that the rate of response was lower amongst those living in urban areas, with lower grades, no upper-secondary education, and those whose parents’ had lower levels of educational attainment.

Information was collected on the egocentric networks of the respondents as well as a wide range of information regarding demographic background characteristics, attitudes, non-cognitive resources, and social and economic resources. The egocentric network data was collected using a name generator question. Each respondent was asked to name the five people that they met and socialized with the most often in their leisure time. It was emphasized to the interviewers that the primary focus was upon friends, but that respondent’s were also able to name parents, brothers, sisters, and other relatives. Respondents were asked to name all five alters before the additional questions about each alter and the relationships between them were asked.

### Outcome variables

The outcome variable for exercise is a binary question regarding whether the ego regularly engages in exercise (1 = yes, 0 = no). The outcome variable for eating is constructed from the question asking the ego about the importance of healthy food. If they responded ‘very important’ or ‘somewhat important’ then this was coded as a 1, and if they responded ‘not important’, then this was coded as a 0.

### Control variables

In each of the models, we include the following control variables: 

• Ethnic background

• Occupational and employment status

• Relationship status

• How many alters the ego named

• How many different settings the ego interacts with the alter across (multiplexity)

• Whether the alter is the same sex as the ego

• Whether the alter is a relative of the ego

• Whether the alter is of the same migration status as the ego (i.e. native Swede, first generation migrant, or second generation migrant)

• Ego-alter tie strength

• Alter health behaviour (either exercising regularly or eating healthily)

The motivation for including these control variables in the models was to try to adjust as much as possible for factors that are associated with both the likelihood of the ego-alter relationship forming, as well as the likelihood of the ego engaging in the health behaviours under study. However, while we do attempt to reduce potential confounding as much as possible, we again re-emphasize that the results still only allow us to observe the association between the health behaviours of the ego and alters.

### Explanatory variables

The key independent variables of interest for the analysis are as follows: 

• Alter health behaviour (either exercising regularly or eating healthily)

• The interaction of the strength of the ego-alter relationship with alter health behaviour (either exercising regularly or eating healthily)

• The interaction of alter type with alter health behaviour (either exercising regularly or eating healthily)

The variable for the strength of the relationship between the ego and alter is based on responses from the ego. The original question was phrased as follows: ‘How good do your think your relationship is?’. Respondents were able to reply on a five point scale, ranging from ‘not at all good’ (1) to ‘very good’ (5). This relationship was coded as strong if the respondent answered with a 5 on the scale, moderate if the respondent answered with a 3 or 4, and weak if the respondent answered with a 1 or 2. The proportion of respondents who did not reply to this question, or who responded that they did not know how good the relationship was, varied according to the number of alters named. Alter type refers to three different classifications. We investigate whether ego-alter homogeneity in terms of gender, migration background, and relative-status increases the likelihood that the ego will engage in the same health behaviours as the alter. Each of these variables indicating ego-alter homogeneity is binary; if the ego and alter are both males, then the variable for gender homogeneity is coded as a 1.

### Statistical analyses

All the results presented here are from dyad-level analyses, meaning that each unit of analysis is a link between the ego and alter, or an ego-alter pair. This means that the number of observations that each respondent to the survey contributes to the analysis varies between one and five, depending on how many alters they named when prompted during the survey collection. We use logistic regressions of the following form to perform the analyses:

$$\log\left(\frac{p_{x}}{1-p_{x}}\right)=\beta_{0}+\beta_{1}X_{1}+\ldots +\beta_{k}X_{k}$$

Where *X*_*k*_ indicates the covariates included in the model, and *β*_*k*_ indicates the corresponding coefficients. Previous research shows that men are more likely to have unhealthy living habits, such as high alcohol consumption, being overweight, smoking, and failing to exercise, than women, though the proportion of men smoking in Sweden has decreased [39]. Given that these gender differences in health behaviours are well documented in Sweden, we will perform separate analyses for males and females. Because we are performing these analyses at the dyad-level, there are multiple observations for each ego. To account for this, we cluster by the identity variable for the ego to account for the non-independence of observations, or the correlation in the error term, giving robust standard errors [40]. The results below are presented in predicted probabilities.

## Results

### Descriptive statistics

The descriptive statistics for the variables can be seen in Table 1. As can be seen, individuals with two Swedish born parents form a higher proportion of the dyads under analysis than individuals of Yugoslavian or Iranian origin. We can also see that the majority of individuals are studying or employed, but that a relatively high proportion is unemployed. We can also see that the majority of individuals are either single or in a non-married relationship. These patterns are not particularly surprising given the age of the individuals under analysis. The majority of respondents named five alters; the mean number of alters named was 4.2, and the median was 5. We can also see that most of the individuals under analysis interacted with their named peers across 2 or 3 different settings, which is shown in the multiplexity variable. We can also see that males name a higher proportion of same-sex alters than do females, while females name a higher proportion of relatives as alters; the proportion of alters who are of the same migration background named by males and females are very similar. Finally, the majority of male ego-alters train regularly, 63%, as do the majority of female ego-alters, 58%. The same is true for eating healthily, with 58% of male ego-alters eating healthily, and 65% of female ego-alters eating healthily.

**Table 1:** Descriptive statistics

		**Male**		**Female**	
**Covariates**	**Categories**	**Dyads**	**Percentage**	**Dyads**	**Percentage**
Ethnic background	Yugoslavia	1,758	31.0	1,568	28.4
	Iran	1,196	21.1	1,196	21.7
	Sweden	2,715	47.9	2,752	49.9
	*Total*	5,669	100.0	5,516	100.0
Occupational /	Studying	2,004	35.4	1,824	33.1
employment status	Employed	1,595	28.1	1,803	32.7
	Studying & employed	470	8.3	905	16.4
	Military	242	4.3	39	0.7
	Unemployed	1,358	24.0	945	17.1
	*Total*	5,669	100.0	5,516	100.0
Civil status	Single	3,945	69.6	3,417	61.9
	Non-married relationship	1,680	29.6	2,055	37.3
	Married	44	0.8	44	0.8
	*Total*	5,669	100.0	5,516	100.0
Number of alters	1	23	0.4	34	0.6
	2	186	3.3	189	3.4
	3	830	14.6	753	13.7
	4	892	15.7	915	16.6
	5	3,738	65.9	3,625	65.7
	*Total*	5,669	100.0	5,516	100.0
Multiplex levels	0	224	4.0	211	3.8
	1	1,125	19.8	1,205	21.8
	2	2,480	43.7	2,780	50.4
	3	1,511	26.7	1,079	19.6
	4	329	5.8	241	4.4
	*Total*	5,669	100.0	5,516	100.0
Alter same sex	Yes	4,967	87.6	4,543	82.4
	No	702	12.4	973	17.6
	*Total*	5,669	100.0	5,516	100.0
Alter relative	Yes	420	7.4	501	9.1
	No	5,249	92.6	5,015	90.9
	*Total*	5,669	100.0	5,516	100.0
Alter same migration background	Yes	3,312	58.4	3,169	57.5
	No	2,357	41.6	2,347	42.5
	*Total*	5,669	100.0	5,516	100.0

Summary statistics showing the number and percentage of ego-alter dyads under analysis.

### Multivariate analyses

**Figure 1:**
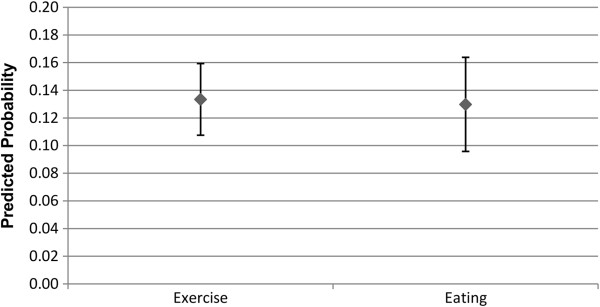
**Males: Alter behaviour on likelihood of ego engaging in behaviour.** The estimated predicted probability reflects how the alter exercising or eating healthily, relative to the alter not exercising regularly or eating healthily, affects the likelihood that the ego will exercise regularly or eat healthily. Confidence intervals are at the 95% confidence level.

**Figure 2:**
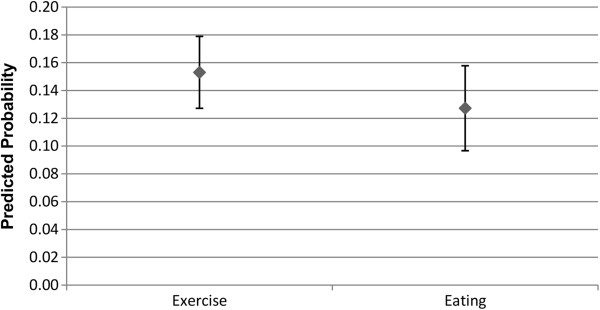
**Females: Alter behaviour on likelihood of ego engaging in behaviour.** The estimated predicted probability reflects how the alter exercising or eating healthily, relative to the alter not exercising regularly or eating healthily, affects the likelihood that the ego will exercise regularly or eat healthily. Confidence intervals are at the 95% confidence level.

Figures 1 and 2 shows the predicted probability for the association between the alter exercising or eating healthily, and the ego exercising or eating healthily. As can be seen, an alter exercising regularly or eating healthily is associated with an increase in the predicted probability that the ego will also exercise regularly or eat healthily, for both males and females. The relative degree of association appears to be greater for exercise than it is for eating healthily, but as the confidence intervals overlap, there is not a statistically significant difference. It can also been seen that the predicted probability estimated is greater for males than it is for females, but again this difference is not statistically significant. As anticipated, this provides support for the first hypothesis.

### Strength of relationship

**Figure 3:**
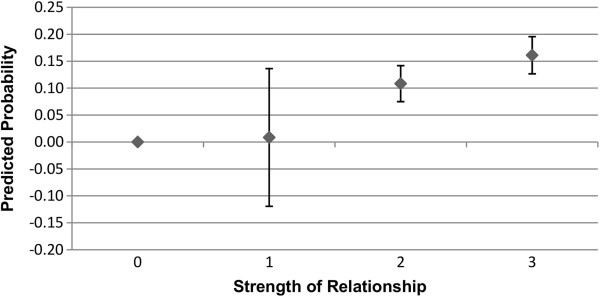
**Males: interaction of strength of relationship with alter behaviour on likelihood of ego exercising regularly.** 0 indicates that the alter does not exercise (0 · 1 ∣ 2 ∣ 3), and is the reference category; 1 indicates that the alter does exercise, and that the strength of the relationship between the ego and alter was weak (1 · 1); 2 indicates that the alter does exercise, and that the strength of the relationship between the ego and alter was moderate (1 · 2); and, 3 indicates that the alter does exercise, and that the strength of the relationship between the ego and alter was strong (1 · 3). Confidence intervals are reported at the 95% confidence level.

**Figure 4:**
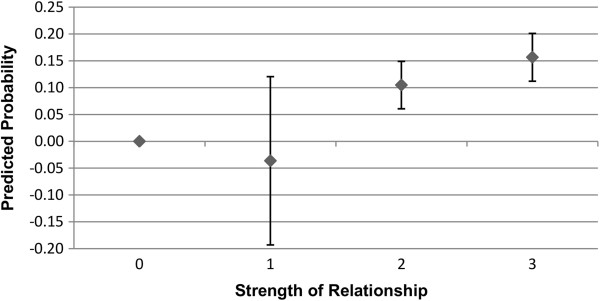
**Males: interaction of strength of relationship with alter behaviour on likelihood of ego eating healthily.** 0 indicates that the alter does not eat healthily (0 · 1 ∣ 2 ∣ 3), and is the reference category; 1 indicates that the alter does eat healthily, and that the strength of the relationship between the ego and alter was weak (1 · 1); 2 indicates that the alter does eat healthily, and that the strength of the relationship between the ego and alter was moderate (1 · 2); and, 3 indicates that the alter does eat healthily, and that the strength of the relationship between the ego and alter was strong (1 · 3). Confidence intervals are reported at the 95% confidence level.

**Figure 5:**
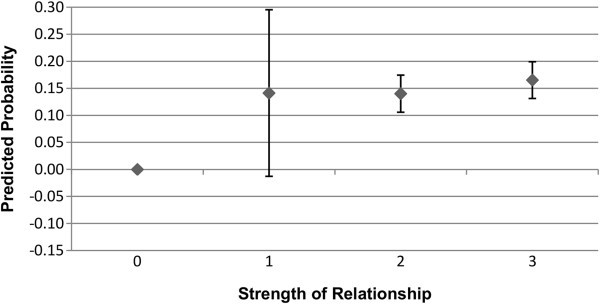
**Females: interaction of strength of relationship with alter behaviour on likelihood of ego exercising regularly.** 0 indicates that the alter does not exercise (0 · 1 ∣ 2 ∣ 3), and is the reference category; 1 indicates that the alter does exercise, and that the strength of the relationship between the ego and alter was weak (1 · 1); 2 indicates that the alter does exercise, and that the strength of the relationship between the ego and alter was moderate (1 · 2); and, 3 indicates that the alter does exercise, and that the strength of the relationship between the ego and alter was strong (1 · 3). Confidence intervals are reported at the 95% confidence level.

**Figure 6:**
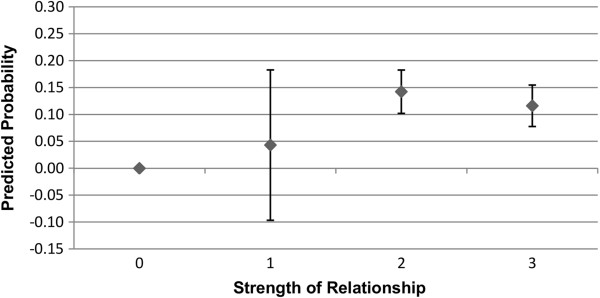
**Females: interaction of strength of relationship with alter behaviour on likelihood of ego eating healthily.** 0 indicates that the alter does not eat healthily (0 · 1 ∣ 2 ∣ 3), and is the reference category; 1 indicates that the alter does eat healthily, and that the strength of the relationship between the ego and alter was weak (1 · 1); 2 indicates that the alter does eat healthily, and that the strength of the relationship between the ego and alter was moderate (1 · 2); and, 3 indicates that the alter does eat healthily, and that the strength of the relationship between the ego and alter was strong (1 · 3). Confidence intervals are reported at the 95% confidence level.

Figures 3, 4, 5, and 6 show the predicted probability for the interaction between the strength of the relationship of the ego with the alter, and the alter exercising or eating healthily. Figures 3 and 4 show these results for males, while Figures 5 and 6 show these results for females. As this alter behaviour-strength of relationship variable is an interaction between a binary variable and a categorical variable with values ranging from 1 to 3, the range from 0 to 3 has the following meaning: 0 indicates that the alter does not exercise or eat healthily (0 · 1 ∣ 2 ∣ 3); 1 indicates that the alter does exercise or eat healthily, and that the strength of the relationship between the ego and alter was reported as weak (1·1); 2 indicates that the alter does exercise or eat healthily, and that the strength of the relationship between the ego and alter was reported as moderate (1 · 2); and, 3 indicates that the alter does exercise or eat healthily, and that the strength of the relationship between the ego and alter was reported as strong (1·3). In Figures 3, 4, 5 and 6, the value 0, where the alter does not exercise or eat healthily, is the reference category.

As can be seen in Figures 3 and 4, relative to the reference category, the relative degree of association of the health behaviours of the alter and the ego increases as the strength of the relationship between the ego and alter increases, though this difference is not statistically significant when the relationship between the ego and alter was weak. The interaction between the strength of the relationship and alter behaviour is associated with a slightly higher predicted probability of the ego engaging in that behaviour for exercise rather than eating healthily, though the difference is small. For females, as shown in Figures 5 and 6, the relative strength of the relationship appears to be less important in terms of the predicted probability of the ego engaging in regular exercise. Even if the relationship was weak, as long as the alter engaged in regular exercise the ego has a substantially elevated probability of exercising regularly. For eating healthily, we see more of a gradient, and that only alters with whom the ego has a moderate or a strong relationship show a statistically significant association in terms of the probability that the ego will also eat healthily. These results for males and females provide support for the second hypothesis.

### Alter type

**Figure 7:**
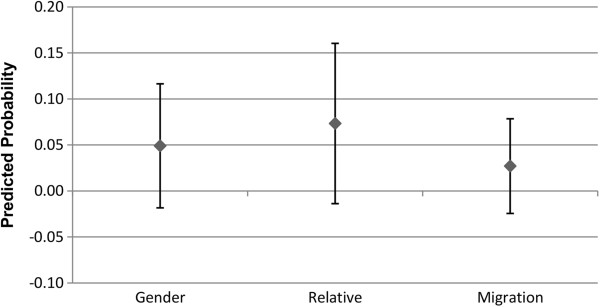
**Males: interaction of alter type with alter behaviour on the likelihood of the ego exercising regularly.** The estimated predicted probability reflects the fact that the ego and alter share the same type [sex, relative status, or ethnic background], and exercise regularly. The reference category in each case are dyads where the alter either is not of the same background type, or does not exercise regularly. Confidence intervals are reported at the 95% confidence level.

**Figure 8:**
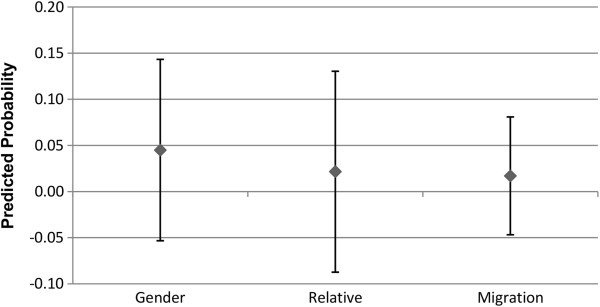
**Males: interaction of alter type with alter behaviour on the likelihood of the ego eating healthily.** The estimated predicted probability reflects the fact that the ego and alter share the same type [sex, relative status, or ethnic background], and eats healthily. The reference category in each case are dyads where the alter either is not of the same background type, or does not eat healthily. Confidence intervals are reported at the 95% confidence level.

**Figure 9:**
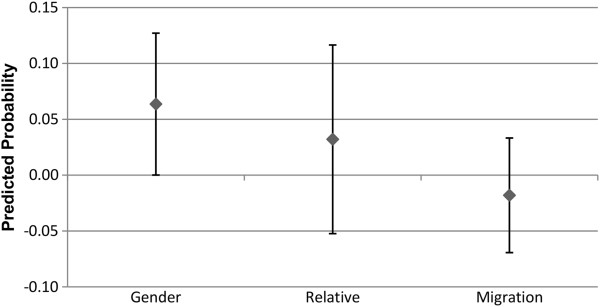
**Females: interaction of alter type with alter behaviour on the likelihood of the ego exercising regularly.** The estimated predicted probability reflects the fact that the ego and alter share the same type [sex, relative status, or ethnic background], and exercise regularly. The reference category in each case are dyads where the alter either is not of the same background type, or does not exercise regularly. Confidence intervals are reported at the 95% confidence level.

**Figure 10:**
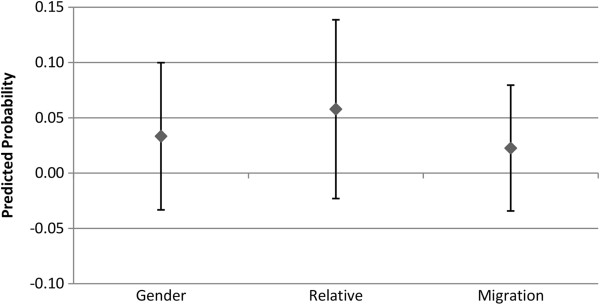
**Females: interaction of alter type with alter behaviour on the likelihood of the ego eating healthily.** The estimated predicted probability reflects the fact that the ego and alter share the same type [sex, relative status, or ethnic background], and eats healthily. The reference category in each case are dyads where the alter either is not of the same background type, or does not eat healthily. Confidence intervals are reported at the 95% confidence level.

Figures 7, 8, 9 and 10 show the predicted probability for the interaction between three different potential characteristics of the alter, and the alter exercising or eating healthily. These three different characteristics are whether the ego and alter are the same gender (1 = yes, 0 = no), whether the alter is a relative of the ego (1 = yes, 0 = no), and whether the alter has the same migration background as the ego (1 = yes, 0 = no). For example, if the ego and alter are both first generation migrants, this will be coded as a 1. As these variables for the relationship between alter behaviour and alter type are interactions between two binary variables, the interaction term consists of the following values: 0 indicates that (a), the alter (i) is not the same gender, (ii) is not a relative, or (iii) does not have the same migration background as the ego, regardless of whether the alter exercises regularly or eats healthily or not. The value 0 may also indicate that the alter does not exercise regularly or eat healthily, even if they are indeed (i) the same gender, (ii) a relative, or (iii) have the same migration background of the ego. So the value 1 indicates that the alter is (i) the same gender, (ii) a relative, or (iii) has the same migration background of the ego, and that they either exercise regularly (Figures 7 and 9), or eat healthily (Figures 8 and 10).

As Figures 7, 8, 9 and 10 indicate, the separate models for males and females did not provide statistically significant support for H3. The third hypothesis had proposed that we would observe a stronger association between the behaviour of the alter and ego in the presence of homophily, but this was not seen for either gender homogeneity, migration status homogeneity, or relative status. Following this, we conducted an additional analysis pooling males and females together. Even in the pooled analysis, the only statistically significant result was that alters who are the same gender as the ego, and who engage in regular exercise, increase the probability that the ego will also engage in regular exercise. No statistically significant results were observed for migration status homogeneity or relative status in regards to exercise propensities, and no statistically significant associations were observed at all for propensities in regards to engaging in healthy eating. These results are available upon request.

## Discussion and conclusions

The results of this study show that the probability of engaging in regular exercise or eating a healthy diet is higher when individuals have friends who also engage in these behaviours. Furthermore, the stronger the relationship between the ego and the alter is, the greater the probability that the ego will behave in a similar way to the alter. These findings are consistent with the broad pattern observed in the social network literature, by which the behaviours observed for connected individuals are persistently correlated. However, the analyses presented above show that the degree to which an ego’s alters are similar to the ego, in terms of being the same gender, or from the same migration background, does not affect the association between the health behaviours of the ego and alters. It was also shown that being a relative of the ego did not affect the association between the ego and alter health behaviours under focus. Nevertheless, when a pooled analysis for males and females was conducted, the results observed were in line with previous findings. These analyses showed that gender homogeneity in the ego-alter relationship showed a statistically significant increase in the probability of the ego and alter behaving similarly in terms of exercising regularly. Given that past studies have also observed that the ego-alter relationship type does affect the degree of association between the health behaviours of alters and the ego, such as smoking and alcohol consumption [10,37], it is possible that the lack of statistical significance in the results for migration status homogeneity and relative-status could therefore be due to a relative lack of power in the statistical analyses.

This study has not been able to identify causality both due to the cross-sectional and observational nature of the data employed, as well as more fundamental problems concerning the identification of peer influence in social network research. Previous research has shown that this causal relationship is not identifiable in the absence of full information concerning both the choice of friends and the proclivity to engage in a given health behaviour [20,22]. While the data used for this study is cross-sectional, these problems of causal identifiability are equally prevalent when analyzing longitudinal datasets, and the use of lagged measures is not an adequate solution [22]. However, while the challenges of identifying causal relationships in social networks from observational data are still being addressed, experiments strongly support the conclusion that peer influence is a real phenomenon [17-19]. Furthermore, sensitivity analyses indicate that the results of studies showing induction of obesity and smoking behaviour ( [10,15], for example) would have to be very seriously confounded to explain away the strongest of the findings [41].

While we are unable to identify causality, we do assume that the exercise and eating behaviour that we investigate in this study have a strong potential for peer transmission because they are visible, and because both exercising and eating are common social activities [27]. Furthermore, the results that we have observed are consistent with the large and growing body of research showing that the behaviours of individuals are associated with the health behaviours of their peers. We have attempted to adjust our estimates for a number of different factors that are likely to influence both the propensity to form friendships as well as to influence the likelihood of engaging in exercise and healthy eating, but there are undoubtedly unobserved factors that we have not been able to adjust for. To be very specific, we have not been able to distinguish between peer influence and assortativity in this study. Even when considered outside of more formal problems of identification [22], it is very difficult to investigate how, for example, exercise behaviour patterns are influenced by peer behaviour, as organized sporting activity, and particularly team sport, serves as a focal point for socialization for many individuals in this age group [28]. Individuals who play team sports engage in exercise themselves, and are more likely than not to also be friends with their teammates. Although it is not possible for us to distinguish between the extent to which the associations that we have observed are due to homophily or an induction effect, they do nevertheless indicate that patterns of eating and exercising are socially clustered. Since these behaviours are socially clustered, individuals who eat a poor diet or exercise infrequently are less likely to adopt a new behaviour, because they are less likely to be connected to individuals who do live more healthy lifestyles [19]. These results points towards potential policy-interventions being targeted towards groups of individuals rather than trying to shape individual behaviour through individualized incentives.

By using egocentric data for this study, we have relied upon the accuracy of the ego in terms of reporting both their own behaviours as well as the behaviours of the alters that they have named. This will obviously lead to a certain degree of measurement error, and this measurement error is likely to be greater when the reported strength of the relationship between the ego and alter is weaker; a weaker relationship is likely to be characterized by, relatively speaking, less intimacy, spending less time together, and having less knowledge about one another, meaning a greater likelihood of lower precision in terms of reporting alter behaviour. Furthermore, by limiting the number of alters that the ego could potentially report to five, the survey procedure will necessarily have restricted the scope of the ego’s full social network that could be captured in the data. As a counterbalance to this limitation, it is probably more likely that the reports on alter behaviour are relatively more accurate than if the ego had had the opportunity to name a greater number of alters, as it is more likely that they would name individuals with whom they had a closer relationship. In addition, past findings suggest that what is important for predicting the association between the ego and alters behaviours is the ego’s perception of the strength of the ego-alter relationship [10,15].

However, despite these factors, this study contributes strongly to what remains a small, though growing, literature concerning the clustering of health behaviours in social networks. In particular, despite research existing on the relationship between social support and exercise adherence, there is only a small body of research concerning the association between peer exercise behaviour and ego exercise behaviour using actual social network data [26-28]. The situation is similar for research concerning eating behaviour, with only a small number of studies addressing this question [26,27,32,42]. Most previous research has been conducted using data from the United States, though a number of these studies have also used data collected in Australia. This study, using a cohort of nineteen year-olds in Sweden, indicates that the patterns of association between ego and alter behaviours that have been observed in samples of adults [10,15,32], as well as samples of young adolescents [26,28], are also present amongst young adults. Furthermore, this study is the first to address this question using data from the Nordic region. It is important to note that this pattern prevails even in a country where the obesity crisis is at a relatively less severe stage of development.

As has been pointed out before, it is very possible that changes in attitudes towards obesity that are influenced by peers could translate into actual changes in weight through different behaviours [15,43]. For example, if a peer abandons smoking, it might not only influence the ego to abandon smoking his or herself, but may also induce a change in attitudes that leads them to adopt a more healthy diet. Future research that addresses parallel changes in a number of different health behaviours in a consistent direction over time would begin to clarify this question. It is important to note that observational data fundamentally restricts the degree to which it is possible to parse out the relative importance of selection effects, exogenous factors, and induction for the transmission of social behaviours [43,44], even with longitudinal data [15]. However, carefully designed experiments have shown that this task is still possible [18,19]. Almost all modern societies face a growing public health dilemma as individuals exercise less and consume a less healthy diet, leading to an increase in the incidence rates of diseases of the circulatory system and cancers. With just cause, concern about the growing number of individuals who are overweight or obese has increased dramatically. While many of these changes are related to shifts in the type of labour in demand due to broader changes to the macroeconomy, and changes to patterns of regular consumption, social influence on health behaviours and changing weight norms are clearly highly salient factors. To develop a greater understanding of the mechanisms by which obesity is spread through a social network, it will be important for future research to continue to go beyond documenting correlations between peers in terms of changes to BMI, and to attempt to elucidate this pattern by investigating the diffusion of actual health behaviours.

## Competing interests

The authors declare that they have no competing interests.

## Authors’ contributions

KJB, CE, and JR conceived and designed the study. KJB performed the statistical analyses. KJB, CE, and JR wrote the manuscript. All authors read and approved the final manuscript.

## Pre-publication history

The pre-publication history for this paper can be accessed here:

http://www.biomedcentral.com/1471-2458/13/784/prepub
